# Interleukin-10 as a potential biomarker reflecting the multidimensional pathological network in patients with severe fever with thrombocytopenia syndrome: viral load, cytokine storm, organ damage, and immune suppression

**DOI:** 10.3389/fimmu.2025.1673091

**Published:** 2026-01-12

**Authors:** Ruihua Zhang, Jingxia Wang, Yanli Xu, Ruize Ma, Wan Peng, Zhouling Jiang, Hongxiao Wu, Chenxi Zhao, Ling Lin, Mengyuan Zhang, Zhihai Chen, Jianping Duan, Yaxian Kong

**Affiliations:** 1 National Key Laboratory of Intelligent Tracking and Forecasting for Infectious Diseases, Beijing Ditan Hospital, Capital Medical University, Beijing, China; 2 Department of Infectious Disease, Beijing Ditan Hospital, Capital Medical University, Beijing, China; 3 Beijing Research Center for Respiratory Infectious Diseases, Beijing, China; 4 National Center for Infectious Diseases, Beijing Ditan Hospital, Capital Medical University, Beijing, China; 5 Department of Infectious Diseases, Yantai Qishan Hospital, Yantai, China; 6 Department of Respiratory Medicine, Binzhou Medical University Hospital, Binzhou, China; 7 Beijing Key Laboratory of Emerging Infectious Diseases, Institute of Infectious Diseases, Beijing Ditan Hospital, Capital Medical University, Beijing, China; 8 Beijing Institute of Infectious Diseases, Beijing, China; 9 Department of Infectious Diseases, Qingdao Sixth People’s Hospital, Shandong, China

**Keywords:** biomarkers, cytokine storm, interleukin-10, prognosis, severe fever with thrombocytopenia syndrome

## Abstract

**Background:**

Severe fever with thrombocytopenia syndrome (SFTS) is a highly fatal infectious disease, potentially driven by cytokine storms. This study evaluates plasma interleukin-10 (IL-10) as a prognostic biomarker by analyzing its association with disease severity and outcomes, aiming to improve early risk stratification and clinical management.

**Methods:**

A retrospective study was conducted on SFTS admitted to Yantai Infectious Disease Hospital from 2022 to 2024. Patients were grouped into survivors (n=160) and non-survivors (n=55). Clinical data from the first 24 hours of admission were collected. To determine independent risk factors influencing prognosis, logistic regression analysis was utilized, particularly assessing the clinical predictive significance of IL-10.

**Results:**

This investigation assessed 215 SFTS patients, where 160 were placed in the survival category and 55 in the mortality category. Multivariate analysis identified IL-10 as an independent predictor of mortality in individuals with SFTS. Receiver Operating Characteristic curve analysis indicated that IL-10 possessed a strong predictive capability for patient mortality, achieving an area under the curve (AUC) of 0.8044 (95% *CI*: 0.7388 - 0.8701; *P* < 0.0001). The optimal cutoff was 29.1 pg/ml (sensitivity 74%, specificity 78%). Furthermore, correlation analysis indicated significant associations between IL-10 and various clinical parameters (all *P* < 0.05), which included SFTS virus RNA, inflammatory markers (such as hypersensitive C-reactive protein and Procalcitonin), signs of multi-organ dysfunction (including Creatine kinase, Lactate dehydrogenase, Aspartate aminotransferase, Blood urea nitrogen, and Creatinine), coagulation irregularities (such as decreased Platelets, prolonged Activated partial thromboplastin time, and elevated D-dimer levels), and indicators of immune suppression (including reduced CD3^+^, CD4^+^, and CD8^+^ T cell counts). Furthermore, IL-10 were found to positively correlate with various pro-inflammatory cytokines including Interleukin-6, Tumor Necrosis Factor-alpha, and Interferon-γ. Patients with IL-10 ≥29.1 pg/ml had higher intensive care unit admission (P<0.0001), SFTS-associated encephalopathy (P = 0.0178), lower viral clearance (P<0.0001) within the first 14 days following symptom onset, and reduced 28-day survival (P = 0.0007). Continuous monitoring of IL-10 showed declining levels in survivors but rising levels in non-survivors.

**Conclusion:**

Plasma IL-10 is an early prognostic biomarker for SFTS, with serial monitoring guiding clinical decisions to improve outcomes.

## 1 Introduction

Severe Fever with Thrombocytopenia Syndrome (SFTS) is an emerging infectious illness attributed to a novel variety of bunyavirus. In 2009, the initial identification of this disease occurred in China (1), and later on, reports of cases emerged from various countries, including the United States (2), South Korea (3), Japan (4), and Vietnam (5). Transmission primarily occurs via tick bites, and recent research has also verified the potential for transmission from animals to humans (6)as well as between humans (7).The disease manifests with diverse clinical presentations. Mild cases primarily exhibit nonspecific symptoms such as fever, fatigue, and gastrointestinal disturbances, along with a reduction in platelet count and a decrease in white blood cells. Severe cases can rapidly progress to life-threatening complications including bleeding tendencies, neurological damage, and even multiple organ failure. Epidemiological data indicate a case fatality rate varies between 5% and 40% (8), highlighting its high lethality risk. Currently, with the continuous rise in both incidence and mortality rates, coupled with the lack of specific therapeutic treatments, SFTS has arisen as a major infectious illness that represents a serious risk to the security of public health in China. There is an urgent need to strengthen prevention and control measures as well as research and development of diagnostic and therapeutic technologies.

Cytokine storm (CS) is a critical pathological mechanism leading to the severe progression of SFTS. The condition is defined by an overactive immune system that initiates a systemic inflammatory response, characterized by a marked rise in the concentrations of pro-inflammatory cytokines (such as tumornecrosisfactor-alpha (TNF-a) and interleukin-6 (IL-6)) in circulation (9, 10). Interleukin-10 (IL-10), a crucial component of the immune regulatory network, demonstrates significant anti-inflammatory characteristics. It is primarily secreted by antigen-presenting cells (including activated T cells, monocytes, B cells, and macrophages) and exerts its immunomodulatory effects by inhibiting the production of pro-inflammatory factors such as TNF-a and IL-6 by macrophages. However, recent studies have found that IL-10 may exhibit pro-inflammatory properties and participate in disease progression under specific pathological conditions, such as in autoimmune diseases, malignant tumors, and coronavirus disease 2019 (COVID-19) infection (11, 12). Furthermore, studies investigating H5N1 influenza virus infection have demonstrated a positive relationship between levels of plasma IL-10 and the viral load present in the throat, suggesting that IL-10 levels may reflect the activity of viral replication (13). Current studies have found that in SFTS patients, plasma IL-10 levels are not only significantly elevated, but also rise earlier than pro-inflammatory factors such as IL-6 (14), making this unique temporal characteristic a potential important biomarker.

Based on the above findings, this study intends to systematically evaluate the correlation between plasma IL-10 concentrations and the severity of disease as well as clinical prognosis in SFTS patients, and to thoroughly explore its predictive efficacy and diagnostic value. This research not only provides a new laboratory indicator for early warning of SFTS, but may also offer a theoretical basis for the development of targeted immunomodulatory treatment strategies.

## 2 Materials and methods

### 2.1 Study population

The clinical data of 249 patients diagnosed with SFTS who were admitted to Yantai Infectious Disease Hospital between January 2022 and December 2024 were analyzed retrospectively in this study. The inclusion criteria were: (1) age≥18 years; (2) laboratory-confirmed SFTS virus infection (including positive virus isolation, positive SFTSV nucleic acid test, or antibody titer increased four-fold in the double sample) (15); (3) admission within 7 days of disease onset and completion of plasma IL-10 testing within 24 hours (The disease onset is defined as the date of the first occurrence of definite clinical symptoms related to the disease determined by reviewing the medical history (fever, fatigue or loss of appetite are the first symptoms to be judged. If the above symptoms are atypical or cannot be recalled, the earliest and more objective gastrointestinal symptoms (such as nausea, vomiting, diarrhea) or neurological symptoms (such as apathy) will be taken as the starting point). Exclusion criteria included: (1) incomplete clinical data; (2) patients with the following underlying conditions: autoimmune diseases, chronic hematological diseases, chronic liver diseases, end-stage renal disease, malignancies, or those with other acute infections within the past month.

### 2.2 Data collection and variables

All data were collected through the hospital’s medical record system and entered into Excel files, with cross-checking performed by two trained researchers. Data collection involves relevant indicators within 24 hours after patient admission, such as demographic data (Age, Gender, Underlying diseases, SFTS-associated encephalopathy (SFTSAE)) and laboratory data (including SFTS virus Ribonucleic acid (SFTSV RNA), White blood cell (WBC), Neutrophil (NEU), Lymphocyte (LYM), Monocyte (MON), Platelet (PLT), Hypersensitive C-reactive protein (Hs-CRP), Procalcitonin (PCT), Alanine aminotransaminase (ALT), Aspartate aminotransferase (AST), Albumin (ALB), Blood urea nitrogen (BUN), Creatinine (CREA), Creatine kinase (CK), Lactate dehydrogenase (LDH), High sensitivity cardiac troponin (Hs-cTn), Activated partial thromboplastin time (APTT), D-dimer, CD3^+^ T cell, CD4^+^ T cell, CD8^+^ T cell, NK cell, B cell, CD4^+^/CD8^+^ ratio, IL-6, IL-10, TNF-a, Interferon-γ (IFN-γ)). The main analysis of this study was based on a single acute phase blood sample collected within 24 hours after admission, aiming to describe the inflammatory state of patients in the initial stage of admission. In a part of patients (63 patients, accounting for 29.3% of the total sample) whose clinical follow-up conditions allowed, we additionally collected a second blood sample within 24 hours before discharge.

SFTSAE refers to neuropsychiatric symptoms (confusion, headaches, dizziness, convulsions, neurological signs, muscle tremors, or coma) in SFTS patients, which cannot be explained by other reasons, and the symptoms are temporally related to the course of SFTS.

Detection of cytokines: The contents of cytokines (IL-6, IL-10, TNF-a and IFN-γ) in plasma were quantitatively detected by multiple microsphere flow immunofluorescence method. The kit used was purchased from Qingdao Raisecare Biotechnology Co., Ltd.

According to the patients’ prognosis, they were classified into two groups: one consisting of those who passed away and another comprising survivors. The deceased group was defined as the patients who died related to SFTS during the hospitalization (the death occurred during the acute hospitalization of SFTS and was clinically judged to be mainly due to SFTS virus infection and its direct complications (such as severe septic shock, multiple organ failure, etc.). Deaths due to clearly unrelated accidental events (such as accidental trauma, acute myocardial infarction, etc.) will be excluded; the survival group comprised patients whose body temperature returned to normal, clinical symptoms improved, and who met the criteria for hospital discharge. For patients who discontinued treatment for personal reasons or were discharged voluntarily, we conducted telephone follow-ups until 28 days after the onset of the disease to confirm their final outcomes.

### 2.3 Ethics approval and consent to participate

This study was conducted in accordance with the principles set forth in the Declaration of Helsinki and obtained approval from the Ethics Committee at Beijing Ditan Hospital, affiliated with Capital Medical University (No. DTEC-KY2022-022-03). The data collection and analysis of this study were carried out under a formal inter agency cooperation framework. As a partner, Yantai Infectious Disease Hospital (data supply center) and Beijing Ditan Hospital (leading research center) have signed a legally effective data sharing and cooperation agreement. The agreement clearly stipulates the purpose, scope, confidentiality terms and the rights and responsibilities of each party for the use of data in this cooperative research. All individuals gave their written informed consent.

### 2.4 Statistical analysis and modeling

The statistical analyses were performed utilizing the software listed below: SPSS version 27.0 (SPSS Inc., Chicago, IL, USA), GraphPad Prism 9.5, R Statistical Software (version 4.4.0; R Core Team 2022), and Zstats v1.0 (available at www.zstats.net).

Data that are normally distributed are presented as mean ± standard deviation (x ± s). For comparisons between groups, an independent sample t-test was employed. Measurements of skewed distribution data are depicted using the median (P25-P75), while the Mann-Whitney U test facilitates comparisons between groups. Categorical variables are represented as ratios, and comparisons between groups are conducted using either the chi-square test or Fisher’s exact probability test. Statistical analyses were performed using a two-tailed method, with significance defined as *P* < 0.05.

In order to identify potential prognostic factors, we first used univariate logistic regression analysis to screen out variables initially associated with survival outcomes. Subsequently, to ensure the robustness of the multifactorial model, we performed multicollinearity diagnosis for all candidate variables. By calculating the variance inflation factor (VIF), it was found that there was collinearity (VIF > 5) between some variables, mainly including LDH and SFTSV RNA, APTT, AST, as well as between CD3 + T cells and CD4 + T cells, CD8 + T cells. Based on clinical significance and statistical correlation, we solved the problem of collinearity by screening representative variables, so that the VIF of each variable finally included in the model was less than 5. In addition, considering the risk of false positives from multiple comparisons, we also used the Benjamini-Hochberg method to correct the false discovery rate (FDR) for the P values of all univariate tests. Finally, the variables with corrected *p* value < 0.05 and collinearity adjustment in univariate analysis were included in the multivariate logistic regression model, and the variables were screened by the reverse stepwise selection method (subject to Akaike information criterion) to identify independent prognostic factors. The predictive capability of each indicator for mortality was assessed using receiver operating characteristic (ROC) analysis. The Youden index was used to determine the optimal cutoff value of IL-10 for predicting the risk of death to achieve an ideal balance between sensitivity and specificity, but it was not applied to the analysis of other biomarkers. To demonstrate how effectively IL-10 can predict disease severity and prognosis in patients with SFTS, Kaplan-Meier survival analysis along with log-rank tests were conducted. Spearman correlation analysis was used to evaluate the association between IL-10 levels and clinical parameters (FDR correction was applied). Given that this study is an exploratory analysis, in order to identify the associations with practical significance, we set the following double criteria to define the significant correlation: the correlation coefficient |*r*| > 0.2 (representing at least a weak to moderate effect size) and the *p* value < 0.05 (representing statistical significance). This criterion aims to avoid paying too much attention to those findings that are statistically significant but the strength of the association is too weak.

## 3 Results

### 3.1 Demographic and general characteristics

**Figure 1:**
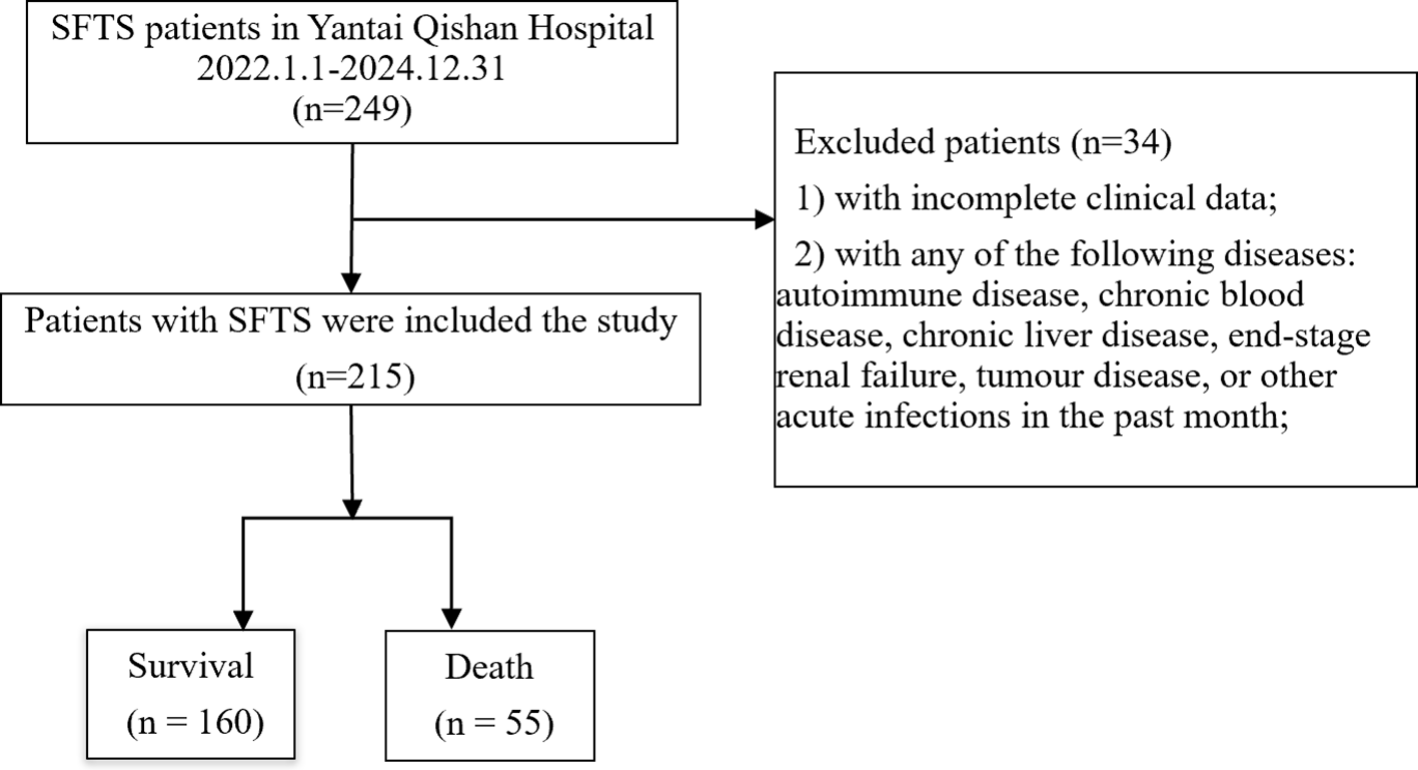
Workflow diagram of recruited and enrolled study participants in study design.

A selection of 249 patients diagnosed with SFTS was made from January 2022 to December 2024 at Yantai Infectious Disease Hospital, adhering to particular inclusion criteria. In total, 215 patients participated in the study cohort, including 55 individuals categorized in the death group, which represented a case fatality rate of 25.58%. **Figure 1** illustrates the study’s flowchart. The median age of the entire patient population was 66 years, while the deceased cohort exhibited a significantly greater median age when compared to the survivors (*P* < 0.001). Among the patients, 105 were male (48.84%), and no notable gender differences were observed between the deceased and surviving groups. In patients with SFTS, common underlying diseases included hypertension (n=56, 26.05%), diabetes (n=37, 17.21%), coronary heart disease (n=9, 4.19%), and cerebrovascular disease (n=7, 3.26%). Nonetheless, the prevalence of these underlying diseases did not show significant differences between the groups of survivors and those who had deceased (**Table 1**). Regarding the main clinical manifestations, the proportion of patients with SFTSAE (*P<*0.001) in the deceased group was significantly higher than that in the survival group (**Table 1**). In relation to laboratory indicators, compared to the survival group, patients in the deceased group had higher levels of SFTSV RNA (15276800.00 (3987840.00, 101680000.00) vs 510880.00 (76876.28, 5580000.00) copies/mL, *P<* 0.001), Hs-CRP (6.27 (2.55, 17.21) vs 2.50 (0.94, 7.58) mg/L, *P<* 0.001), PCT (0.53 (0.16, 0.95) vs 0.12 (0.08, 0.28) ng/ml, *P<* 0.001), AST (191.60 (126.05, 421.95) vs 119.05 (63.37, 236.65) U/L, *P<* 0.001), BUN (8.65 (5.88, 12.85) vs 5.84 (4.55, 7.46) mmol/L, *P<* 0.001), CREA (78.10 (60.00, 120.00)vs 66.00 (53.22, 78.25) umol/L, *P=* 0.002), CK (814.00 (322.00, 1363.00)vs 387.50 (184.00, 1072.25) U/L, *P=* 0.006), LDH (772.00 (567.00, 1643.00)vs 500.00 (356.00, 812.25) U/L, *P<* 0.001), Hs-cTn (94.00 (46.40, 265.60)vs 28.75 (13.45, 67.00) ng/L, *P<* 0.001), APTT (53.30 (47.75, 65.55)vs 48.10 (41.98, 53.65) s, *P<* 0.001), D-dimer (5510.00 (2815.00, 11305.00)vs 2370.00 (1355.00, 4162.50) µg/ml, *P<* 0.001), NK cell (205.00 (129.00, 393.00)vs 172.50 (106.50, 278.50) cells/μL, *P=* 0.016), B cell (143.00 (63.00, 335.00)vs 103.00 (62.00, 195.00) cells/μL, *P* = 0.044), IL-6 (90.90 (29.50, 199.35)vs 13.30 (5.18, 34.75) pg/ml, *P* < 0.001), IL-10 (58.80 (30.00, 121.65)vs 10.70 (3.98, 29.83) pg/ml, *P* < 0.001), TNF-a (1.80 (1.15, 3.00)vs 1.50 (0.91, 2.20) pg/ml, *P* = 0.031), and IFN-γ (265.90 (125.85, 607.05)vs 93.85 (28.00, 235.75) pg/ml, *P<* 0.001) upon admission, while PLT (51.00 (38.50, 69.50)vs 68.50 (52.75, 93.00) *10^^9^/L, *P<* 0.001), ALB (30.74 ± 4.87 vs 33.43 ± 4.88 g/L, *P<* 0.001), CD3^+^ T cell (223.00 (167.50, 296.50) vs 357.50 (230.00, 599.00) cells/μL, *P<* 0.001), CD4^+^ T cell (85.00 (65.00, 135.50) vs 168.50 (102.00, 294.25) cells/μL, *P<* 0.001), CD8^+^ T cell (110.00 (66.00, 167.50) vs 164.00 (96.75, 260.00) cells/μL, *P* = 0.002), and CD4^+^/CD8^+^ ratio (0.90 (0.52, 1.38) vs 1.10 (0.72, 1.60), *P* = 0.024) were lower. No notable distinctions were detected in the other remaining indicators when comparing the two groups (**Table 1**).

**Table 1:** Clinical characteristics and laboratory parameters compared between the survival and death group in SFTS patients.

Variables	All (n=215)	Survival (n=160)	Death (n=55)	*P*
Age^a^ (year)	66.00 (60.00, 73.00)	65.00 (59.00, 71.00)	71.00 (64.00, 77.00)	<0.001
Gender, n (%)				0.965
Male	105 (48.84)	78 (48.75)	27 (49.09)	
Female	110 (51.16)	82 (51.25)	28 (50.91)	
Underlying diseases, n (%)				
Diabetes, n (%)	37 (17.21)	25 (15.62)	12 (21.82)	0.294
Hypertension, n (%)	56 (26.05)	45 (28.12)	11 (20.00)	0.236
Coronary Heart Disease, n (%)	9 (4.19)	7 (4.38)	2 (3.64)	1.000
Cerebrovascular disease, n (%)	7 (3.26)	6 (3.75)	1 (1.82)	0.798
SFTSAE, n (%)	122 (56.74)	82 (51.25)	40 (72.73)	0.006
SFTSV RNA^a^ (copies/mL)	1329280.00 (156046.56, 13739200.00)	510880.00 (76876.28, 5580000.00)	15276800.00 (3987840.00, 101680000.00)	<0.001
WBC^a^ (*10^^9^/L)	2.10 (1.46, 3.36)	2.15 (1.48, 3.50)	2.02 (1.45, 2.86)	0.452
NEU ^a^ (*10^^9^/L)	1.29 (0.87, 2.05)	1.29 (0.84, 2.02)	1.35 (0.97, 2.35)	0.510
LYM^a^ (*10^^9^/L)	0.45 (0.32, 0.69)	0.49 (0.32, 0.72)	0.44 (0.30, 0.55)	0.125
MON^a^ (*10^^9^/L)	0.14 (0.08, 0.34)	0.15 (0.09, 0.34)	0.11 (0.06, 0.33)	0.091
PLT^a^ (*10^^9^/L)	66.00 (47.00, 85.00)	68.50 (52.75, 93.00)	51.00 (38.50, 69.50)	<0.001
Hs-CRP^a^ (mg/L)	3.24 (1.24, 8.89)	2.50 (0.94, 7.58)	6.27 (2.55, 17.21)	<0.001
PCT ^a^ (ng/ml)	0.17 (0.09, 0.47)	0.12 (0.08, 0.28)	0.53 (0.16, 0.95)	<0.001
ALT^a^ (U/L)	66.20 (40.90, 115.15)	67.15 (36.38, 115.95)	65.70 (48.85, 109.00)	0.409
AST^a^ (U/L)	130.10 (72.35, 271.95)	119.05 (63.37, 236.65)	191.60 (126.05, 421.95)	<0.001
ALB^b^ (g/L)	32.74 ± 5.01	33.43 ± 4.88	30.74 ± 4.87	<0.001
BUN^a^ (mmol/L)	6.17 (4.77, 8.64)	5.84 (4.55, 7.46)	8.65 (5.88, 12.85)	<0.001
CREA^a^ (umol/L)	68.00 (54.00, 84.10)	66.00 (53.22, 78.25)	78.10 (60.00, 120.00)	0.002
CK^a^ (U/L)	488.00 (204.50, 1158.50)	387.50 (184.00, 1072.25)	814.00 (322.00, 1363.00)	0.006
LDH ^a^ (U/L)	583.00 (387.00, 960.50)	500.00 (356.00, 812.25)	772.00 (567.00, 1643.00)	<0.001
Hs-cTn^a^ (ng/L)	41.50 (17.50, 108.75)	28.75 (13.45, 67.00)	94.00 (46.40, 265.60)	<0.001
APTT^a^ (s)	49.40 (42.95, 56.25)	48.10 (41.98, 53.65)	53.30 (47.75, 65.55)	<0.001
D-dimer ^a^ (µg/ml)	2600.00 (1490.00, 5975.00)	2370.00 (1355.00, 4162.50)	5510.00 (2815.00, 11305.00)	<0.001
CD3^+^ T cell^a^ (cells/μL)	304.00 (210.50, 567.50)	357.50 (230.00, 599.00)	223.00 (167.50, 296.50)	<0.001
CD4^+^ T cell^a^ (cells/μL)	139.00 (83.50, 264.00)	168.50 (102.00, 294.25)	85.00 (65.00, 135.50)	<0.001
CD8^+^ T cell^a^ (cells/μL)	143.00 (88.00, 232.00)	164.00 (96.75, 260.00)	110.00 (66.00, 167.50)	0.002
NK cell^a^ (cells/μL)	182.00 (111.50, 298.50)	172.50 (106.50, 278.50)	205.00 (129.00, 393.00)	0.016
B cell^a^ (cells/μL)	106.00 (62.00, 219.00)	103.00 (62.00, 195.00)	143.00 (63.00, 335.00)	0.044
CD4^+^/CD8^+^ ratio^a^	1.07 (0.67, 1.51)	1.10 (0.72, 1.60)	0.90 (0.52, 1.38)	0.024
IL-6^a^ (pg/ml)	21.80 (8.15, 54.50)	13.30 (5.18, 34.75)	90.90 (29.50, 199.35)	<0.001
IL-10^a^ (pg/ml)	18.00 (5.65, 50.45)	10.70 (3.98, 29.83)	58.80 (30.00, 121.65)	<0.001
TNF-a^a^ (pg/ml)	1.50 (1.00, 2.35)	1.50 (0.91, 2.20)	1.80 (1.15, 3.00)	0.031
IFN-γ^a^ (pg/ml)	128.90 (38.80, 337.70)	93.85 (28.00, 235.75)	265.90 (125.85, 607.05)	<0.001

^a^By means of the nonparametric test, expressed as median (P25-P75), M is the median, P25 is the lower quartile, P75 is the upper quartile. ^b^By means of the parametric test, expressed as mean ± standard deviation (SD). SFTS, Severe fever with thrombocytopenia syndrome; SFTSAE, SFTS associated encephalopathy; SFTSV RNA, SFTS virus Ribonucleic acid; WBC, White blood cell; NEU,Neutrophil; LYM, Lymphocyte; MON, Monocyte; PLT, Platelet; Hs-CRP, Hypersensitive C-reactive protein; PCT, Procalcitonin; ALT, Alanine aminotransaminase; AST, Aspartate aminotransferase; ALB, Albumin; BUN, Blood urea nitrogen; CREA, Creatinine; CK, Creatine kinase; LDH, Lactate dehydrogenase; Hs-cTn, High sensitivity cardiac troponin; APTT, Activated partial thromboplastin time; IL-6, Interleukin-6; IL-10, Interleukin-10; TNF-a, Tumornecrosisfactor-alpha; IFN-γ, Interferon-γ.

### 3.2 Mortality risk factors in patients with SFTS

Upon examining the clinical traits and lab metrics of the patients, a univariate logistic regression analysis indicated that factors such as age, SFTSAE, SFTSV RNA, PLT, AST, ALB, BUN, CREA, LDH, APTT, D-dimer, CD3^+^ T cell, CD4^+^ T cell, CD8^+^ T cell, NK cell, B cell, CD4^+^/CD8^+^ ratio, IL-6, IL-10, TNF-a, and IFN-γ could potentially be linked to mortality outcomes. Following this, these factors with corrected *p* value < 0.05 and collinearity adjustment in univariate analysis were incorporated into a multifactorial logistic regression analysis. The results showed that elevated levels of Age (*OR*:1.052; 95%*CI*:1.007-1.100; *P* = 0.022), IL-10 (*OR*:1.010; 95%*CI*:1.002-1.019; *P* = 0.020), IL-6 (*OR*:1.004; 95%*CI*:1.001-1.008; *P* = 0.011), and NK cell (*OR*:1.003; 95%*CI*:1.001-1.005; *P* = 0.005), as well as decreased levels of CD4^+^ T cell (*OR*:0.991; 95%*CI*:0.986-0.997; *P* = 0.002), were identified as independent risk factors contributing to mortality among patients with SFTS (**Table 2**).

**Table 2:** Univariate and multivariate analysis of factors associated with fatal outcome among patients with SFTS.

Variables	Univariate analysis		Multivariate analysis	
	*P*	*OR (*95%*CI)*	*P*	*OR (*95%*CI)*
Age	<0.001	1.065 (1.029 ~ 1.102)	0.022	1.052 (1.007 ~ 1.100)
Gender	0.965	0.986 (0.534 ~ 1.821)		
Diabetes	0.296	1.507 (0.698 ~ 3.252)		
Hypertension	0.239	0.639 (0.303 ~ 1.346)		
Coronary Heart Disease	0.814	0.825 (0.166 ~ 4.094)		
Cerebrovascular disease	0.496	0.475 (0.056 ~ 4.038)		
SFTSAE	0.006	2.537 (1.299 ~ 4.955)		
SFTSV RNA	0.002	1.001 (1.001 ~ 1.001)		
WBC	0.546	0.951 (0.806 ~ 1.121)		
NEU	0.765	0.971 (0.803 ~ 1.175)		
LYM	0.086	0.446 (0.177 ~ 1.122)		
MON	0.833	1.078 (0.536 ~ 2.166)		
PLT	<0.001	0.974 (0.960 ~ 0.987)		
Hs-CRP	0.051	1.016 (1.000 ~ 1.033)		
PCT	0.170	1.023 (0.990 ~ 1.057)		
ALT	0.506	1.001 (0.998 ~ 1.003)		
AST	0.010	1.001 (1.001 ~ 1.002)		
ALB	0.001	0.897 (0.841 ~ 0.957)		
BUN	<0.001	1.195 (1.105 ~ 1.292)		
CREA	<0.001	1.011 (1.005 ~ 1.018)		
CK	0.325	1.000 (1.000 ~ 1.000)		
LDH	<0.001	1.001 (1.001 ~ 1.001)		
Hs-cTn	0.079	1.000 (1.000 ~ 1.001)		
APTT	<0.001	1.044 (1.021 ~ 1.067)		
D-dimer	<0.001	1.001 (1.001 ~ 1.001)		
CD3^+^ T cell	<0.001	0.997 (0.996 ~ 0.999)		
CD4^+^ T cell	<0.001	0.993 (0.989 ~ 0.996)	0.002	0.991 (0.986 ~ 0.997)
CD8^+^ T cell	0.026	0.997 (0.995 ~ 0.999)		
NK cell	0.005	1.002 (1.001 ~ 1.004)	0.005	1.003 (1.001 ~ 1.005)
B cell	0.002	1.003 (1.001 ~ 1.005)		
CD4^+^/CD8^+^ ratio	0.022	0.549 (0.329 ~ 0.917)		
IL-6	<0.001	1.006 (1.003 ~ 1.009)	0.011	1.004 (1.001 ~ 1.008)
IL-10	<0.001	1.021 (1.014 ~ 1.029)	0.020	1.010 (1.002 ~ 1.019)
TNF-a	0.046	1.123 (1.002 ~ 1.258)		
IFN-γ	<0.001	1.001 (1.001 ~ 1.002)		

SFTS, Severe fever with thrombocytopenia syndrome; SFTSAE, SFTS associated encephalopathy; SFTSV RNA, SFTS virus Ribonucleic acid; WBC, White blood cell; NEU,Neutrophil; LYM, Lymphocyte; MON, Monocyte; PLT, Platelet; Hs-CRP, Hypersensitive C-reactive protein; PCT, Procalcitonin; ALT, Alanine aminotransaminase; AST, Aspartate aminotransferase; ALB, Albumin; BUN, Blood urea nitrogen; CREA, Creatinine; CK, Creatine kinase; LDH, Lactate dehydrogenase; Hs-cTn, High sensitivity cardiac troponin; APTT, Activated partial thromboplastin time; IL-6, Interleukin-6; IL-10, Interleukin-10; TNF-a, Tumornecrosisfactor-alpha; IFN-γ, Interferon-γ.

### 3.3 IL-10 as a biomarker for mortality risk assessment in SFTS

**Figure 2:**
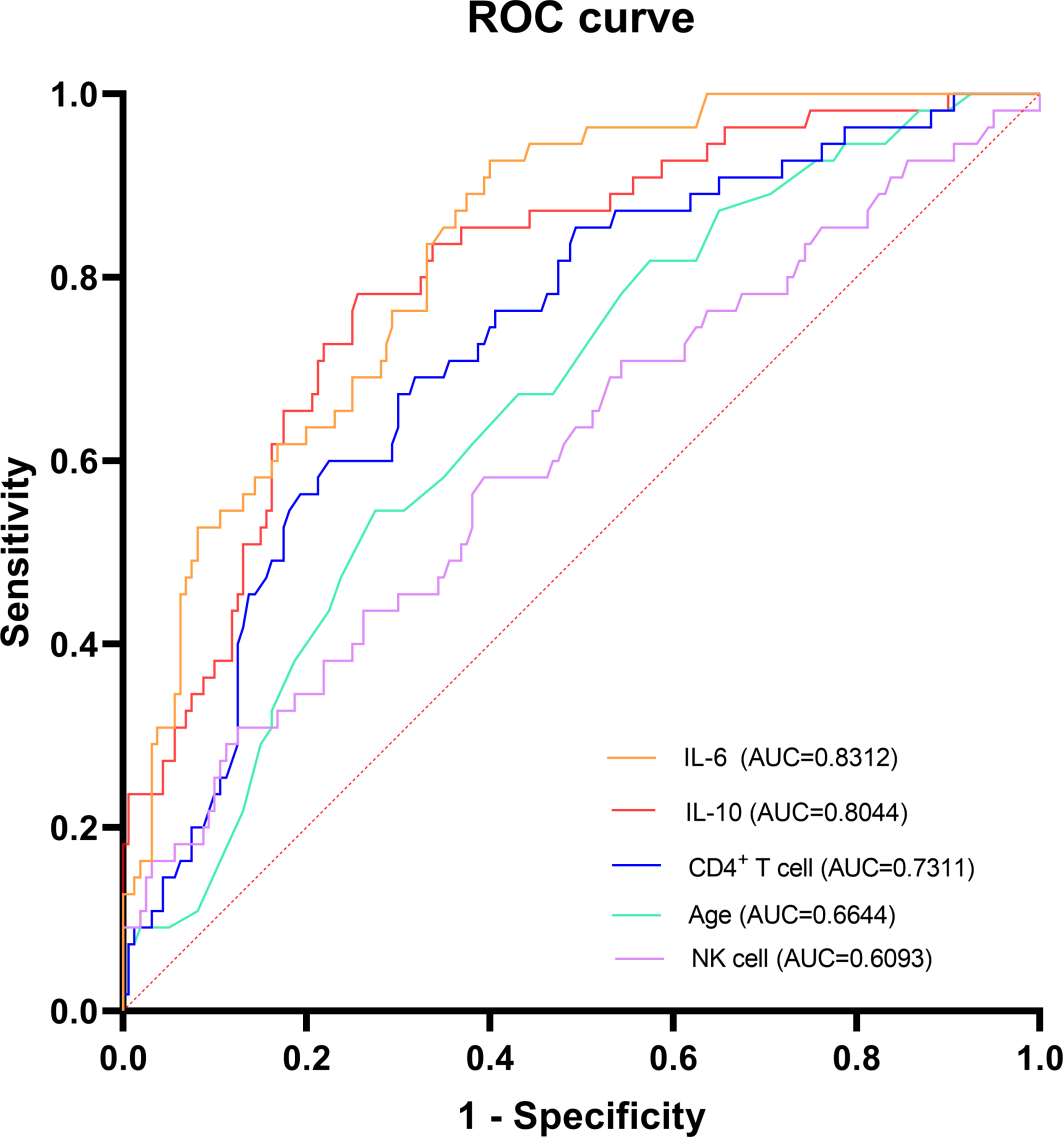
Receiver operating characteristic (ROC) curves analysis of prognostic indicators for the mortality of SFTS patients. SFTS, Severe fever with thrombocytopenia syndrome; IL-6, Interleukin-6; IL-10, Interleukin-10.

To evaluate the diagnostic capability of IL-10 in predicting fatal outcomes in SFTS patients, its predictive performance was compared against other established independent risk factors. As illustrated in **Figure 2**, the area under the curve (AUC) for IL-10 was found to be 0.8044 (95% *CI*: 0.7388 - 0.8701; *P* < 0.0001), for IL-6 was 0.8312 (95%*CI*: 0.7747-0.8877; *P* < 0.0001), for CD4^+^ T cell was 0.7311 (95%*CI*: 0.6559-0.8063; *P* < 0.0001), for Age was 0.6644 (95%*CI*: 0.5847-0.7440; *P* = 0.0003), and for NK cell was 0.6093 (95%*CI*: 0.5198-0.6988; *P* = 0.0157) (**Table 3**). The findings presented that IL-10 showed good discriminative ability in distinguishing the prognosis of patients, and its potential as an auxiliary prognostic evaluation index is worthy of attention. The ideal cut-off threshold for IL-10, as established by the highest Youden index, is 29.1 pg/ml, featuring a sensitivity of 74% (95%*CI*: 68%**-**81%) and a specificity of 78% (95%*CI*: 67%-89%) (refer to **Table 3**).

**Table 3:** Predictive value of prognostic indicators for the mortality of SFTS patients.

Biomarkers	AUC	95% *CI*	Value of *P*	Cut-off value	Sensitivity % (95% *CI*)	Specificity % (95% *CI*)
IL-6	0.8312	0.7747 - 0.8877	<0.0001	18.8	60 (52 - 68)	93 (86 - 100)
IL-10	0.8044	0.7388 -0.8701	<0.0001	29.1	74 (68 - 81)	78 (67 - 89)
CD4^+^ T cell	0.7311	0.6559 - 0.8063	<0.0001	99.5	23 (16 - 29)	40 (27 - 53)
Age	0.6644	0.5847 -0.7440	0.0003	70.5	72 (66 - 79)	55 (41 - 68)
NK cell	0.6093	0.5198 -0.6988	0.0157	197.5	61 (53 - 68)	58 (45 - 71)

SFTS, Severe fever with thrombocytopenia syndrome; IL-10, Interleukin-10; IL-6, Interleukin-6.

### 3.4 The impact of different levels of IL-10 on SFTS patients

Based on the cut-off value of 29.1 pg/ml, all patients were divided into a low IL-10 group (<29.1 pg/ml) and a high IL-10 group (≥29.1 pg/ml). A comparison of demographic characteristics and laboratory parameters between the two groups (**Table 4**) revealed that, compared to the low IL-10 group, patients in the high IL-10 group had higher levels of SFTSV RNA (15180080.00 (4493760.00, 51956000.00) vs 240560.00 (49848.00, 1277200.00) copies/mL, *P* < 0.001), Hs-CRP (6.54 (2.42, 13.87) vs 2.27 (0.83, 5.63) mg/L, *P<* 0.001), PCT (0.41 (0.16, 0.77) vs 0.11 (0.07, 0.24) ng/ml, *P<* 0.001), ALT (78.65 (53.05, 142.35) vs 54.50 (31.95, 112.05) U/L, *P=* 0.001), AST (216.45 (128.75, 347.35) vs 97.30 (55.85, 170.95)U/L, *P<* 0.001), BUN (7.96 (5.37, 11.21) vs 5.82 (4.62, 7.34) mmol/L, *P<* 0.001), CREA (75.50 (61.00, 101.50) vs 65.00 (52.95, 76.00) umol/L, *P<* 0.001), CK (871.50 (417.00, 2150.25)vs 315.00 (159.00, 816.00)U/L, *P<* 0.001), LDH (960.50 (630.00, 1565.50)vs 427.00 (314.50, 645.50) U/L, *P<* 0.001), Hs-cTn (76.15 (36.60, 263.75) vs 25.00 (11.25, 65.50) ng/L, *P* < 0.001), APTT (55.80 (50.40, 65.22) vs 46.00 (40.60, 50.35)s, *P<* 0.001), D-dimer (5245.00 (2600.00, 11695.00) vs 2090.00 (1165.00, 2840.00)µg/ml, *P* < 0.001), IL-6 (50.65 (20.93, 149.05)vs 12.80 (5.05, 31.15) pg/ml, *P* < 0.001), TNF-a (1.90 (1.10, 3.00) vs 1.40 (0.90, 2.20) pg/ml, *P* = 0.002), and IFN-γ (227.35 (115.12, 535.80) vs 79.50 (22.35, 208.25) pg/ml, *P* < 0.001) upon admission. Conversely, the high IL-10 group exhibited lower levels of LYM (0.39 (0.31, 0.53) vs 0.53 (0.36, 0.78) *10^^9^/L, *P* < 0.001), MON (0.11 (0.06, 0.33) vs 0.16 (0.10, 0.32) *10^^9^/L, *P=*0.013), PLT (51.00 (39.75, 67.00) vs 74.00 (58.00, 97.00) *10^^9^/L, *P<* 0.001), ALB (30.60 ± 4.36 vs 34.11 ± 4.93 g/L, *P* < 0.001), CD3^+^ T cells (238.00 (172.00, 320.50) vs 412.00 (246.50, 682.50) cells/μL, *P<* 0.001), CD4^+^ T cells (98.50 (68.00, 140.25) vs 184.00 (113.50, 327.50) cells/μL, *P<* 0.001), CD8^+^ T cells (106.50 (67.00, 169.50) vs 175.00 (108.00, 276.50) cells/μL, *P<* 0.001), and CD4^+^/CD8^+^ ratio (0.92 (0.54, 1.25) vs 1.10 (0.72, 1.65), *P=* 0.015). More importantly, patients belonging to the high IL-10 category had a significantly higher risk of developing SFTSAE (p<0.001), being transferred to the intensive care unit (ICU) (*P<*0.001), and experiencing mortality (*P<*0.001) after the onset of the disease.

**Table 4:** Clinical characteristics and laboratory parameters of patients with SFTS, according the IL-10 cut off value on admission.

Variables	All (n=215)	IL-10^low^ (<29.1) (n=131)	IL-10^high^ (≥29.1) (n=84)	*P*
Outcome, n(%)	55 (25.58)	12 (9.16)	43 (51.19)	<0.001
ICU adimission, n(%)	103 (47.91)	36 (27.48)	67 (79.76)	<0.001
Age^a^ (year)	66.00 (60.00, 73.00)	65.00 (59.00, 71.50)	68.00 (61.00, 75.25)	0.053
Gender, n (%)				0.398
Male	105 (48.84)	67 (51.15)	38 (45.24)	
Female	110 (51.16)	64 (48.85)	46 (54.76)	
Underlying diseases, n (%)				
Diabetes, n (%)	37 (17.21)	21 (16.03)	16 (19.05)	0.567
Hypertension, n (%)	56 (26.05)	33 (25.19)	23 (27.38)	0.721
Coronary Heart Disease, n (%)	9 (4.19)	5 (3.82)	4 (4.76)	1.000
Cerebrovascular disease, n (%)	7 (3.26)	5 (3.82)	2 (2.38)	0.853
SFTSAE, n (%)	122 (56.74)	62 (47.33)	60 (71.43)	<0.001
SFTSV RNA^a^ (copies/mL)	1329280.00 (156046.56, 13739200.00)	240560.00 (49848.00, 1277200.00)	15180080.00 (4493760.00, 51956000.00)	<0.001
WBC^a^ (*10^^9^/L)	2.10 (1.46, 3.36)	2.21 (1.57, 3.54)	1.93 (1.35, 3.11)	0.097
NEU ^a^ (*10^^9^/L)	1.29 (0.87, 2.05)	1.30 (0.83, 1.99)	1.27 (0.89, 2.30)	0.958
LYM^a^ (*10^^9^/L)	0.45 (0.32, 0.69)	0.53 (0.36, 0.78)	0.39 (0.31, 0.53)	<0.001
MON^a^ (*10^^9^/L)	0.14 (0.08, 0.34)	0.16 (0.10, 0.32)	0.11 (0.06, 0.33)	0.013
PLT^a^ (*10^^9^/L)	66.00 (47.00, 85.00)	74.00 (58.00, 97.00)	51.00 (39.75, 67.00)	<0.001
Hs-CRP^a^ (mg/L)	3.24 (1.24, 8.89)	2.27 (0.83, 5.63)	6.54 (2.42, 13.87)	<0.001
PCT ^a^ (ng/ml)	0.17 (0.09, 0.47)	0.11 (0.07, 0.24)	0.41 (0.16, 0.77)	<0.001
ALT^a^ (U/L)	66.20 (40.90, 115.15)	54.50 (31.95, 112.05)	78.65 (53.05, 142.35)	0.001
AST^a^ (U/L)	130.10 (72.35, 271.95)	97.30 (55.85, 170.95)	216.45 (128.75, 347.35)	<0.001
ALB^b^ (g/L)	32.74 ± 5.01	34.11 ± 4.93	30.60 ± 4.36	<0.001
BUN^a^ (mmol/L)	6.17 (4.77, 8.64)	5.82 (4.62, 7.34)	7.96 (5.37, 11.21)	<0.001
CREA^a^ (umol/L)	68.00 (54.00, 84.10)	65.00 (52.95, 76.00)	75.50 (61.00, 101.50)	<0.001
CK^a^ (U/L)	488.00 (204.50, 1158.50)	315.00 (159.00, 816.00)	871.50 (417.00, 2150.25)	<0.001
LDH ^a^ (U/L)	583.00 (387.00, 960.50)	427.00 (314.50, 645.50)	960.50 (630.00, 1565.50)	<0.001
Hs-cTn^a^ (ng/L)	41.50 (17.50, 108.75)	25.00 (11.25, 65.50)	76.15 (36.60, 263.75)	<0.001
APTT^a^ (s)	49.40 (42.95, 56.25)	46.00 (40.60, 50.35)	55.80 (50.40, 65.22)	<0.001
D-dimer ^a^ (µg/ml)	2600.00 (1490.00, 5975.00)	2090.00 (1165.00, 2840.00)	5245.00 (2600.00, 11695.00)	<0.001
CD3^+^ T cell^a^ (cells/μL)	304.00 (210.50, 567.50)	412.00 (246.50, 682.50)	238.00 (172.00, 320.50)	<0.001
CD4^+^ T cell^a^ (cells/μL)	139.00 (83.50, 264.00)	184.00 (113.50, 327.50)	98.50 (68.00, 140.25)	<0.001
CD8^+^ T cell^a^ (cells/μL)	143.00 (88.00, 232.00)	175.00 (108.00, 276.50)	106.50 (67.00, 169.50)	<0.001
NK cell^a^ (cells/μL)	182.00 (111.50, 298.50)	182.00 (113.50, 275.00)	179.50 (111.75, 324.50)	0.566
B cell^a^ (cells/μL)	106.00 (62.00, 219.00)	102.00 (60.50, 201.50)	124.00 (67.75, 270.00)	0.133
CD4^+^/CD8^+^ ratio^a^	1.07 (0.67, 1.51)	1.10 (0.72, 1.65)	0.92 (0.54, 1.25)	0.015
IL-6^a^ (pg/ml)	21.80 (8.15, 54.50)	12.80 (5.05, 31.15)	50.65 (20.93, 149.05)	<0.001
TNF-a^a^ (pg/ml)	1.50 (1.00, 2.35)	1.40 (0.90, 2.20)	1.90 (1.10, 3.00)	0.002
IFN-γ^a^ (pg/ml)	128.90 (38.80, 337.70)	79.50 (22.35, 208.25)	227.35 (115.12, 535.80)	<0.001

^a^By means of the nonparametric test, expressed as median (P25-P75), M is the median, P25 is the lower quartile, P75 is the upper quartile. ^b^By means of the parametric test, expressed as mean ± standard deviation (SD). SFTS, Severe fever with thrombocytopenia syndrome; SFTSAE, SFTS associated encephalopathy; SFTSV RNA, SFTS virus Ribonucleic acid; ICU, Intensive care unit; WBC, White blood cell; NEU,Neutrophil; LYM, Lymphocyte; MON, Monocyte; PLT, Platelet; Hs-CRP, Hypersensitive C-reactive protein; PCT, Procalcitonin; ALT, Alanine aminotransaminase; AST, Aspartate aminotransferase; ALB, Albumin; BUN, Blood urea nitrogen; CREA, Creatinine; CK, Creatine kinase; LDH, Lactate dehydrogenase; Hs-cTn, High sensitivity cardiac troponin; APTT, Activated partial thromboplastin time; IL-6, Interleukin-6; IL-10, Interleukin-10; TNF-a, Tumornecrosisfactor-alpha; IFN-γ, Interferon-γ.

### 3.5 Correlation between IL-10 and laboratory parameters

**Figure 3:**
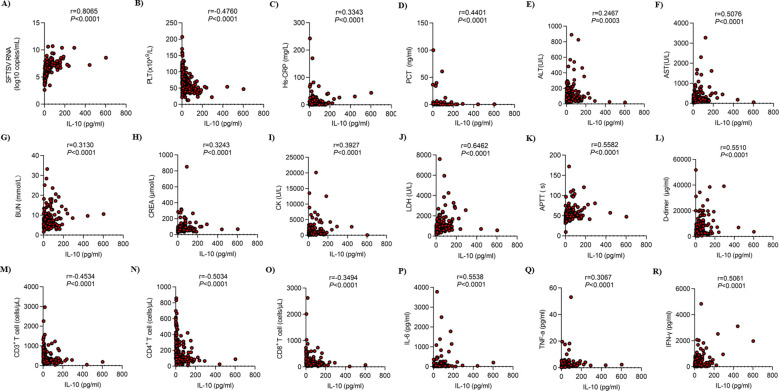
Correlation between IL-10 and laboratory parameters in SFTS patients. Correlation between IL-10 and **(A)**SFTSV RNA, **(B)** PLT, **(C)** Hs-CRP, **(D)** PCT, **(E)** ALT, **(F)** AST, **(G)** BUN, **(H)** CREA, **(I)** CK, **(J)** LDH, **(K)** APTT, **(L)** D-dimer, **(M)** CD3^+^ T cell, **(N)** CD4^+^ T cell, **(O)** CD8^+^ T cell, **(P)** IL-6, **(Q)** TNF-a, **(R)** IFN-γ in SFTS patients. SFTS, Severe fever with thrombocytopenia syndrome; IL-10, Interleukin-10; SFTSV RNA, SFTS virus Ribonucleic acid; PLT, Platelet; Hs-CRP, Hypersensitive C-reactive protein; PCT, Procalcitonin; ALT, Alanine aminotransaminase; AST, Aspartate aminotransferase; BUN, Blood urea nitrogen; CREA, Creatinine; CK, Creatine kinase; LDH, Lactate dehydrogenase; APTT, Activated partial thromboplastin time; IL-6, Interleukin-6; TNF-a, Tumornecrosisfactor-alpha; IFN-γ, Interferon-γ.

To enhance our understanding of IL-10, we subsequently conducted a correlation analysis between IL-10 and SFTSV RNA, multi-organ function (heart, liver, kidney), coagulation function, immune function, and cytokine levels (**Figure 3**). The results indicated a strong positive relationship between IL-10 and SFTSV RNA (r=0.8065, *P* < 0.0001), as well as a moderate positive correlation with AST (r=0.5076, *P* < 0.0001), LDH (r=0.6462, *P* < 0.0001), APTT (r=0.5582, *P* < 0.0001), D-dimer (r=0.5510, *P* < 0.0001), IL-6 (r=0.5538, *P* < 0.0001), and IFN-γ (r=0.5061, *P* < 0.0001), and mildly positively correlated with Hs-CRP (r=0.3343, *P* < 0.0001), PCT (r=0.4401, *P* < 0.0001), BUN (r=0.3130, *P* < 0.0001), CREA (r=0.3243, *P* < 0.0001), CK (r=0.3927, *P* < 0.0001), and TNF-a (r=0.3067, *P* < 0.0001). However, it showed a moderate negative correlation with CD4^+^ T cells (r=-0.5034, *P* < 0.0001), and mild negative correlations with PLT (r=-0.4760, *P* < 0.0001), CD3^+^ T cells (r=-0.4534, *P* < 0.0001), and CD8^+^ T cells (r=-0.3494, *P* < 0.0001). The findings suggest that the IL-10 levels align with clinical functional metrics and may represent the body’s overall state.

### 3.6 IL-10 is associated with the severity of the disease and poor prognosis in SFTS patients

**Figure 4:**
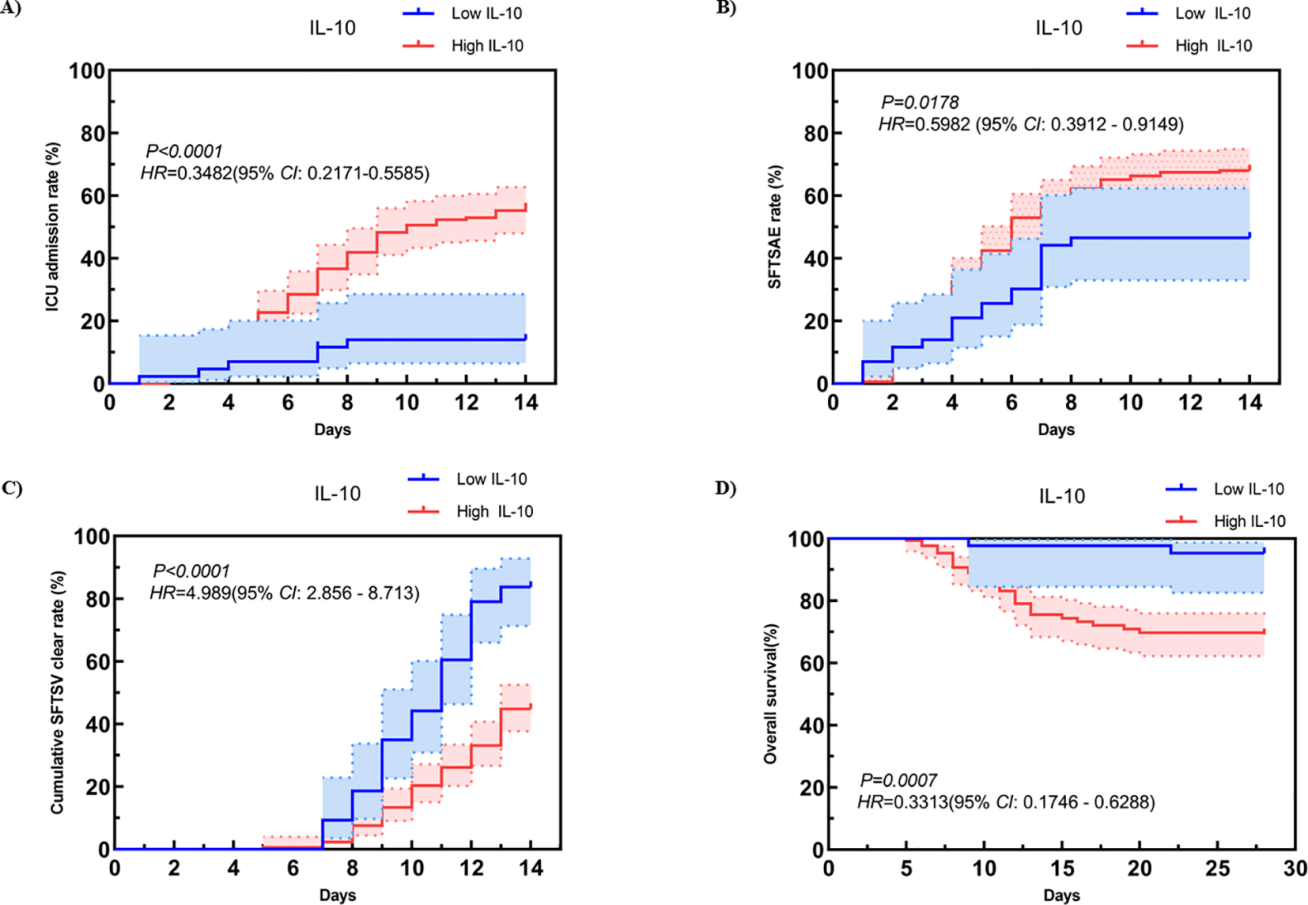
Comparison of **(A)** ICU admission rate within 14 days after onset, **(B)** SFTSAE incidence rate within 14 days after onset, **(C)** Cumulative SFTSV clear rate within 14 days after onset and **(D)** survival rate within 28 days after onset between high IL-10 level group and low IL-10 level group in SFTS patients. SFTS, Severe fever with thrombocytopenia syndrome. SFTSAE, SFTS associated encephalopathy; SFTSV, SFTS virus; ICU, Intensive care unit; IL-10, Interleukin-10.

To assess the diagnostic importance of IL-10 in individuals with SFTS, we performed several Kaplan-Meier curve analyses. The findings showed that, in comparison to the low IL-10 group, individuals in the high IL-10 group experienced a notably higher risk of requiring ICU admission within 14 days of symptom onset (*HR*: 0.3482, 95% *CI*: 0.2171-0.5585, *P* < 0.0001, **Figure 4A**), a significantly elevated incidence of SFTSAE (*HR*: 0.5982, 95% *CI*: 0.3912-0.9149*, P* = 0.0178, **Figure 4B**), and cumulative rate of virus clearance was markedly lower in this group (*HR*: 4.989, 95% *CI*: 2.856-8.713, *P* < 0.001, **Figure 4C**). Additionally, the 28-day survival rate was significantly reduced (*HR*: 0.3313, 95% *CI:* 0.1746-0.6288*, P* = 0.0007, **Figure 4D**). This further highlights the relationship between increased IL-10 levels and the severity of the disease, along with negative outcomes in patients with SFTS.

### 3.7 Dynamic changes in IL-10 levels in the survival and mortality groups of patients

**Figure 5:**
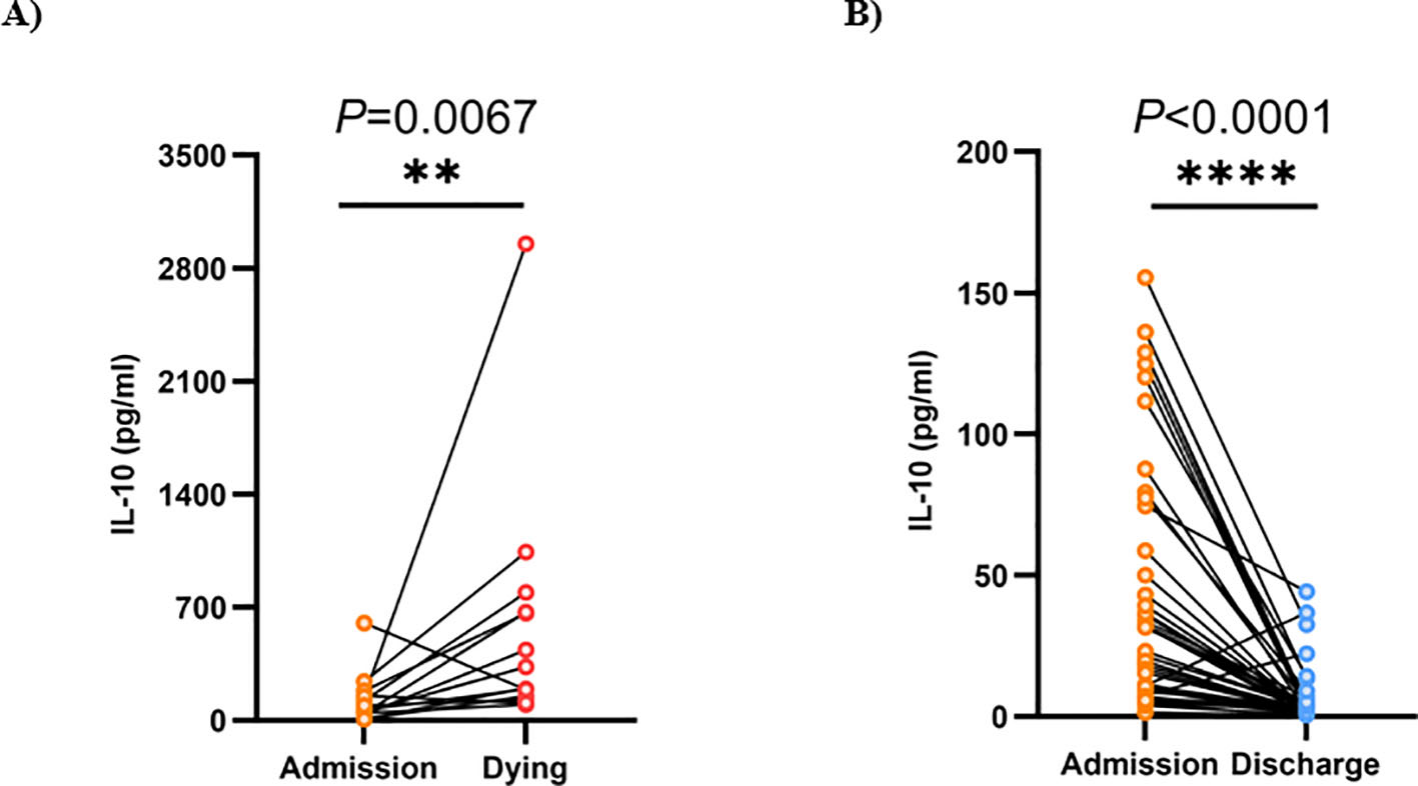
Dynamic changes of IL-10 level in admission and follow-up of SFTS patients. **(A)** Dynamic changes of IL-10 level in admission (within 24 hours after admission) and Dying (within 24 hours before discharge) of SFTS non-survivors (n = 14). **(B)** Dynamic changes of IL-10 level in SFTS surviving patients at admission (within 24 hours after admission) and discharge (within 24 hours before discharge) (n = 49). SFTS, Severe fever with thrombocytopenia syndrome; IL-10, Interleukin-10. Statistical analysis using Wilcoxon matched pairs signed rank test. Exclusion of patients with single IL-10 test from the analysis. **p < 0.01; ****p < 0.0001.

To gain a deeper understanding of the correlation between IL-10 levels and the prognosis of patients with SFTS, we monitored the fluctuations of IL-10 levels during the initial (within 24 hours after admission) and final (within 24 hours before discharge) blood tests throughout hospitalization for both the survival and non-survival groups. Our findings indicated that in the non-survival group (n=14), the IL-10 levels rose as the disease advanced in the majority of patients (76.65 (43.22, 150.75) vs 264.70 (159.38, 667.55) pg/ml, *P* = 0.0067, **Figure 5A**). Conversely, in the survivor group (n=49), IL-10 levels significantly decreased as the patients’ condition improved and the SFTSV nucleic acid test turned negative (21.20 (9.50, 50.10) vs 2.20 (1.60, 4.10) pg/ml, *P* < 0.0001, **Figure 5B**).

## 4 Discussion

SFTS represents a newly recognized infectious illness characterized by a high mortality rate, presenting a considerable risk to public health worldwide. Currently, the complete understanding of its pathogenesis remains unclear, and the CS is considered a key pathological mechanism leading to severe SFTS. This study, by analyzing the relationship between plasma IL-10 levels and laboratory parameters, disease severity, and adverse outcomes in SFTS patients, confirmed that IL-10 may act as a viable biomarker that comprehensively reflects the multidimensional pathological process of “viral load-inflammatory storm-organ damage-immune suppression” in SFTS patients, while also providing a new intervention target for the targeted treatment of SFTS.

**Figure 6:**
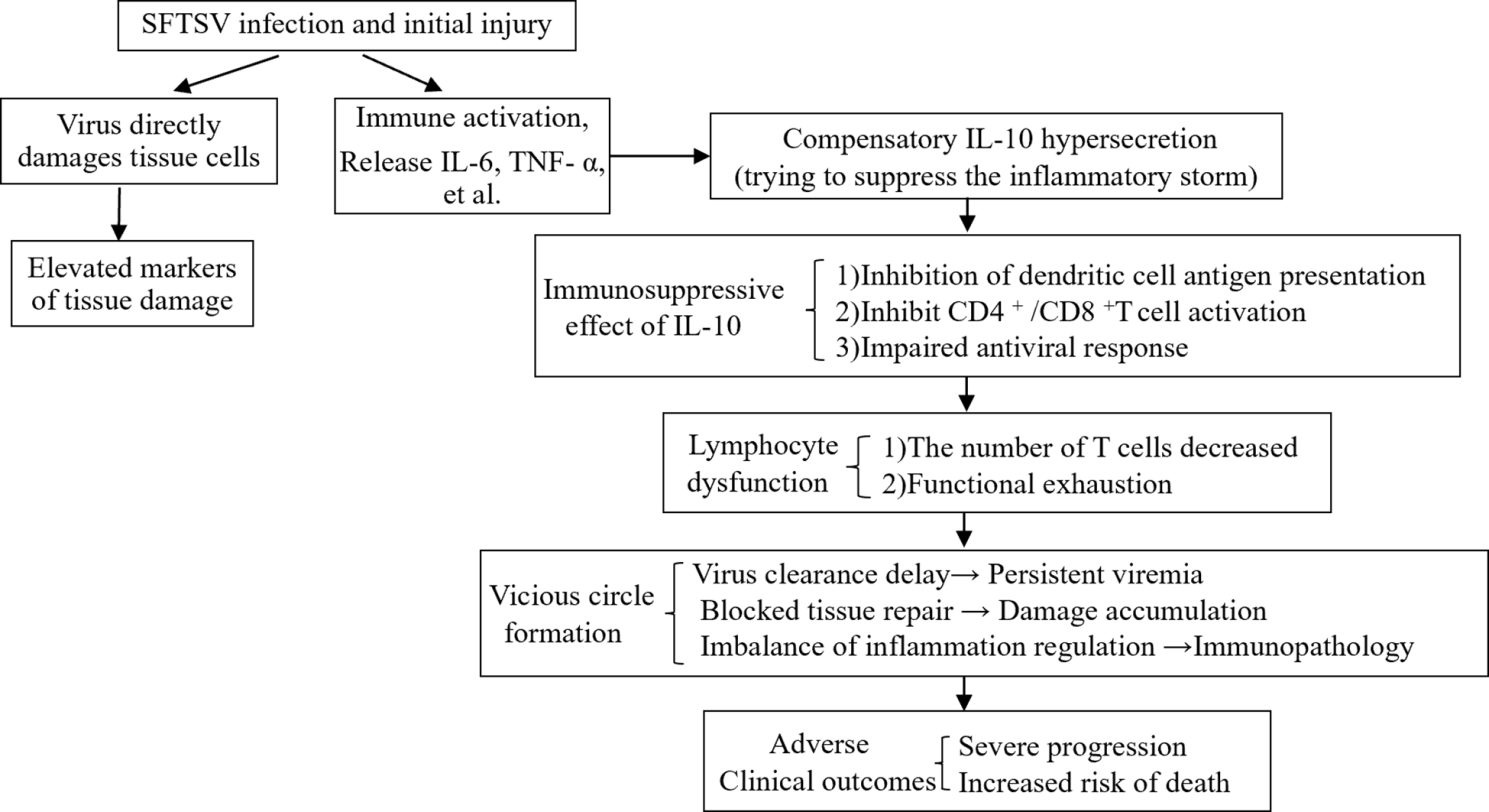
Pathogenic role model of IL-10 in patients with SFTS. SFTS, Severe fever with thrombocytopenia syndrome; SFTSV, SFTS virus; IL-10, Interleukin-10; IL-6, Interleukin-6; TNF-a, Tumornecrosisfactor-alpha.

IL-10, which functions as a pleiotropic cytokine, is mainly generated by activated mononuclear macrophages, regulatory T cells, and B lymphocytes. The variation in its expression levels profoundly reflects the state of host’s specific immune response against SFTS virus infection. During the initial phases of the illness, a moderate increase in IL-10 actually serves an essential protective function. By inhibiting excessively active inflammatory responses, IL-10 can effectively prevent tissue damage caused by cytokine storms, a regulatory function that is essential for maintaining immune homeostasis (16). However, when IL-10 expression continues to be abnormally elevated, it transforms from a protective factor into a pathogenic factor, leading to adverse consequences. In certain autoimmune diseases and cancers in human patients, it has been identified as playing a pathological role as an immune-activating and pro-inflammatory cytokine. While treating Crohn’s disease using recombinant human interleukin-10 (rhIL-10), Tilg et al. discovered unexpectedly that it stimulated the production of the pro-inflammatory cytokine IFN-γ, revealing the complexity of IL-10 in the regulation of the human immune system and explaining the limited clinical efficacy of the treatment (11). Naing and colleagues found that pegylated IL-10, also known as Pegilodecakin, has the potential to systematically stimulate immune responses in individuals with cancer, notably improving the function of CD8^+^ T cells and encouraging the expansion of polyclonal T cells (17). Additionally, in specific viral infections, an overproduction of IL-10 might obstruct the elimination of pathogens and facilitate the advancement of the disease. Huang et al. measured plasma cytokines in patients with COVID-19 and found that ICU patients exhibited markedly elevated levels of IL-10 in their plasma when contrasted with those in non-ICU settings (18). Han et al. discovered that serum IL-10 levels were significantly higher in the critical group than in the moderate and severe groups among COVID-19 patients, and that IL-10 levels were positively correlated with CRP levels, further indicating that IL-10 could predict the severity of disease in COVID-19 patients (19). Redford et al. innovatively revealed the dual regulatory role of IL-10 in Mycobacterium tuberculosis infection, discovering that IL-10 exerts a protective immune effect in the initial phase of infection, while in the chronic phase, it transitions to a suppressive role by regulating macrophage polarization and T cell exhaustion (20). In addition, in the regulation of T cell subsets, IL-10 can inhibit the function of effector T cells. IL-10 directly inhibits the activation and proliferation of CD4^+^ and CD8^+^ T cells through JAK1/STAT3 signaling pathway (21–23), which is manifested by the negative correlation between the number of T cell subsets and the level of IL-10. IL-10 is also a key effector molecule of Treg cells (24, 25). These cells further inhibit effector T cell function by secreting IL-10 (26, 27), forming a negative feedback loop. Interestingly, in the pathological process of SFTS, changes in plasma IL-10 levels also demonstrate significant clinical implications. Our findings indicate a significant rise in plasma IL-10 concentrations in the group of individuals who passed away. While other studies also view IL-10 as a predictive indicator of SFTS severity, they have not performed a comprehensive examination of the clinical significance of IL-10 (14, 28, 29). In contrast, our research reveals a significant positive correlation between plasma IL-10 levels and the viral load in the peripheral blood of patients, and patients with high IL-10 levels face a significantly increased risk of multi-organ damage. We consider the reason for this occurrence to be that in the initial phases of SFTS infection, the elevation of IL-10 may be a negative feedback mechanism by which the body attempts to suppress excessive inflammatory responses. However, as the endogenous production of IL-10 continues to increase, we hypothesize that it could serve as both an immune activator and pro-inflammatory agent, thereby enhancing the release of additional mediators involved in the cytokine storm. This situation resembles the hyperinflammatory response associated with viral sepsis seen in individuals suffering from human endotoxemia and patients with severe or critical COVID-19. The abnormal elevation of IL-10 may not only fail to effectively inhibit IL-6, but may be accompanied by or lead to the insufficient production of transforming growth factor-β (TGF-β) with anti-inflammatory and immune regulatory functions. In addition, In the regulation of T cell subsets, excessive IL-10 secretion can cause a decrease in the number of lymphocytes and inhibit the function of effector T cells, and multiple factors together aggravate the immune imbalance and inflammatory injury. It is this imbalanced regulation that leads to an imbalance in the immune response, which neither effectively clears the virus nor may trigger excessive inflammatory damage (12, 30) (**Figure 6**). However, the specific mechanisms by which IL-10 contributes to pathological damage in SFTS patients still require further exploration.

Regarding the clinical application value of IL-10, this study has evaluated from multiple aspects and found that IL-10 levels have high predictive value for SFTS patients. Firstly, we conducted logistic regression analysis and found that increased levels of IL-10 represent a standalone risk factor for mortality among patients with SFTS. The analysis of the ROC curve revealed that IL-10 is a good predictor of unfavorable outcomes in SFTS patients upon admission. Additionally, we conducted a comparison of clinical and laboratory tests between groups exhibiting low and high levels of IL-10. The findings demonstrated that individuals in the high IL-10 group were older, exhibited a greater viral load at admission, had a significantly higher occurrence of SFTSAE, and faced more severe dysfunction in the cardiac, hepatic, renal, coagulation, and immune systems. Subsequently, we assessed the relationship between IL-10 levels and various laboratory parameters, including SFTSV RNA, inflammatory markers (Hs-CRP, PCT), cardiac (CK, LDH), hepatic (ALT, AST), renal (BUN, CREA), and coagulation (PLT, APTT, D-dimer) function, as well as immune cell subsets (CD3^+^, CD4^+^, CD8^+^ T cells) and cytokine profiles (TNF-a, IFN-γ,IL-6). It was found that IL-10 levels are consistent with clinical functional indicators and can serve as a biomarker that comprehensively reflects the multidimensional pathological process of “viral load-inflammatory storm-organ damage-immune suppression” in SFTS patients.

We applied the optimal cutoff value of IL-10 (29.1 pg/ml) to categorize patients into low IL-10 and high IL-10 groups. According to the Kaplan-Meier survival curve analysis, compared to patients in the low IL-10 group, those in the high IL-10 group exhibited a notably higher risk of ICU admission and incidence of SFTSAE, reduced cumulative virus clearance rate within 14 days after onset, and a notably decreased 28-day survival rate. We also monitored IL-10 levels in certain survivors as well as in patients who ultimately did not survive the disease, observing that IL-10 levels in those who passed away rose as the disease advanced, whereas levels in surviving patients steadily approached normal ranges as their condition improved. Based on these findings, dynamic monitoring of IL-10 level changes holds significant clinical value: on one hand, it can serve as an objective indicator for assessing disease severity, and on the other hand, it can provide a reliable laboratory basis for prognosis judgment. Furthermore, from the perspective of translational medicine, our research also presents promising therapeutic prospects for targeted intervention in the IL-10 signaling pathway in SFTS patients. Potential strategies include using IL-10 receptor antagonists to block excessively activated signal transduction, or restoring the balance between IL-10 and other cytokines through precise immune modulation. These innovative therapies are expected to become breakthroughs in improving the prognosis of severe SFTS patients. However, additional preclinical research and clinical trials remain necessary to confirm their safety and effectiveness. It should be noted that SFTSV is known to have significant geographical and genetic heterogeneity, which can be mainly divided into multiple genotypes. Different genotypes may have differences in transmission efficiency, cell tropism and pathogenicity (31, 32). All cases in this study were from a single center in Shandong Province, China. The specific immune response pattern characterized by elevated IL-10 observed by the Institute mainly reflects the host response triggered by the local epidemic strains. At present, data on how different SFTSV genotypes specifically regulate host immune responses (especially IL-10 pathway) are still very limited. When our results are applied to other regions where SFTSV with different genotypes are prevalent, its universality needs to be carefully verified. Future multicenter studies should integrate viral genomics and host immunology analysis to clarify the association between viral genetic variation and specific immune phenotypes (such as IL-10 levels), which is of great significance for developing immune intervention strategies with broad applicability.

This research presents several limitations. Firstly, the conclusions drawn may have limited external validity due to the small sample size and the retrospective design conducted at a single center. Future validation through multicenter prospective studies is necessary. Second, the diagnosis of SFTSAE is based solely on clinical manifestations and exclude diagnosis, with a lack of data on cerebrospinal fluid (routine, biochemical, and etiology) and head imaging examinations. Therefore, future research needs to combine objective indicators such as neuroimaging and cerebrospinal fluid analysis to establish a more accurate diagnostic system. Third, due to the limitations of the current clinical data, this study was limited to measuring IL-10 levels only at admission and discharge in a subset of surviving and deceased patients, and we cannot provide the results of the TGF-β. In the future research, we will systematically detect the level of IL-10, TGF-β and IL-6, longitudinally analyze the dynamic changes and signaling pathways of them. Consequently, it is advisable to carry out further multicenter cohort studies involving larger sample sizes and incorporating long-term follow-up to improve the validation of our study results.

## 5 Conclusion

This research is the first to confirm through multidimensional analysis that plasma IL-10 is a reliable predictive indicator for early detection of high-risk SFTS patients. Continuously monitoring changes in IL-10 levels not only effectively assesses the risk of disease progression but may also provide an important foundation for determining the timing of clinical interventions and the formulation of individualized treatment strategies, thereby improving patient prognosis. This discovery offers a novel potential target for the monitoring and precision therapy of SFTS.

## Data Availability

The original contributions presented in the study are included in the article/supplementary material. Further inquiries can be directed to the corresponding author/s.

## Glossary

SFTS: Severe fever with thrombocytopenia syndrome
IL-10: Interleukin-10
AUC: Area under the curve
ICU: Intensive care unit
SFTSAE: SFTS-associated encephalopathy
SFTSV: SFTS virus
CS: Cytokine storm
TNF-a: Tumornecrosisfactor-alpha
IL-6: Interleukin-6
COVID-19: Coronavirus disease 2019
SFTSV RNA: SFTS virus Ribonucleic acid
WBC: White blood cell
NEU: Neutrophil
LYM: Lymphocyte
MON: Monocyte
PLT: Platelet
Hs-CRP: Hypersensitive C-reactive protein
PCT: Procalcitonin
ALT: Alanine aminotransaminase
AST: Aspartate aminotransferase
ALB: Albumin
BUN: Blood urea nitrogen
CREA: Creatinine
CK: Creatine kinase
LDH: Lactate dehydrogenase
Hs-cTn: High sensitivity cardiac troponin
APTT: Activated partial thromboplastin time
IFN-γ: Interferon-γ
VIF: Variance inflation factor
FDR: False discovery rate
ROC: Receiver operating characteristic
rhIL-10: Recombinant human interleukin-10
TGF-β: Transforming growth factor-β
